# Monosexual Cercariae of *Schistosoma japonicum* Infection Protects Against DSS-Induced Colitis by Shifting the Th1/Th2 Balance and Modulating the Gut Microbiota

**DOI:** 10.3389/fmicb.2020.606605

**Published:** 2021-01-05

**Authors:** Hongli Zhou, Xiaojing Zeng, Dongchen Sun, Zhe Chen, Weixin Chen, Liwei Fan, Yanin Limpanont, Paron Dekumyoy, Wanchai Maleewong, Zhiyue Lv

**Affiliations:** ^1^Joint Program of Pathobiology, Fifth Affiliated Hospital, Zhongshan School of Medicine, Sun Yat-sen University, Guangzhou, China; ^2^NHC Key Laboratory of Control of Tropical Diseases, Hainan Medical University, Haikou, China; ^3^Faculty of Tropical Medicine, Mahidol University, Bangkok, Thailand; ^4^Faculty of Medicine, Khon Kaen University, Khon Kaen, Thailand; ^5^Department of Laboratory Medicine, The First Affiliated Hospital, Hainan Medical University, Haikou, China

**Keywords:** inflammatory bowel disease, monosexual cercariae, *Schistosoma japonicum*, Th1/Th2, gut microbiota

## Abstract

Inflammatory bowel disease (IBD)-related inflammation is closely associated with the initiation and progression of colorectal cancer. IBD is generally treated with 5-aminosalicylic acid and immune-modulating medication, but side effects and limitations of these therapies are emerging. Thus, the development of novel preventative or therapeutic approaches is imperative. Here, we constructed a dextran sodium sulphate (DSS)-induced IBD mouse model that was infected with monosexual *Schistosoma japonicum* cercariae (mSjci) at day 1 or administered dexamethasone (DXM) from days 3 to 5 as a positive control. The protective effect of mSjci on IBD mice was evaluated through their assessments of their clinical signs, histopathological lesions and intestinal permeability. To uncover the underlying mechanism, the Th1/Th2 balance and Treg cell population were also examined. Additionally, the alterations in the gut microbiota were assessed to investigate the interaction between the mSjci-modulated immune response and pathogenic microbiome. Mice treated with DSS and mSjci showed fewer IBD clinical signs and less impaired intestinal permeability than DSS-treated mice. Mechanistically, mSjci modulated the Th1/Th2 balance by repressing IFN-γ production, promoting IL-10 expression and enhancing the Treg subset population. Moreover, mSjci notably reshaped the structure, diversity and richness of the gut microbiota community and subsequently exerted immune-modulating effects. Our findings provide evidence showing that mSjci might serve as a novel and effective protective strategy and that the gut microbiota might be a new therapeutic target in IBD.

## Introduction

As a chronic inflammatory condition of the gastrointestinal tract characterized by abdominal pain, diarrhea, rectal bleeding and weight loss (Abraham and Cho, 2009; Zhang and Li, 2014; Yu and Rodriguez, 2017), inflammatory bowel disease (IBD) affects millions of people worldwide, results in a substantial financial cost each year (Burisch et al., 2013; Kaplan, 2015), and has become a global health issue with increasing incidence and prevalence (Molodecky et al., 2012; Kaplan, 2015). Over the past several decades, IBD therapies have been rapidly developed. At present, the commonly recommended therapies include 5-aminosalicylic acid, corticosteroids, immune modulators, and monoclonal antibodies against TNF-α (such as infliximab) (Baumgart and Sandborn, 2007; Triantafillidis et al., 2011). However, although these therapies can effectively alleviate IBD symptoms, side effects and limitations, including immunosuppression, drug resistance and tremendous expenses, have gradually emerged and restricted the constant application of these therapies (Ben-Horin and Chowers, 2011; Marchetti and Liberato, 2014). Moreover, IBD-related inflammation is closely associated with the initiation and progression of colorectal cancer. Therefore, the development of a novel protective or therapeutic approach for IBD-related inflammation is urgently needed. Elucidation of the underlying pathogenesis of IBD would undoubtedly provide new insights that will contribute to the discovery of novel protective approaches. IBD is conventionally known as a consequence of an aberrant inflammatory state in the intestinal mucosa that involves enhanced infiltration and activation of immune cells (Di Sabatino et al., 2012; Zhang and Li, 2014), which leads to abnormal expression of inflammatory molecules, such as tumor necrosis factor α (TNF-α), interferon-γ (IFN-γ) and interleukin-23 (IL-23) (Park et al., 2017). Based on this finding, traditional therapies targeting inflammatory cytokines have been developed, but these therapeutics have gradually exhibited increased side effects and limitations.

Recent studies have shown that IBD is closely associated with alterations in the gut microbiota diversity and disruptions in the balance between commensal and potentially pathogenetic microbiome constituents (Gkouskou et al., 2014; Goto et al., 2015; Ni et al., 2017). For example, decreases in the Firmicutes abundance and increases in the Proteobacteria abundance have been observed in the intestinal tract of IBD patients (Khan et al., 2019). Moreover, the cumulative body of evidence implies that IBD might arise from aberrant immune responses to the gut microbiota (Abraham and Cho, 2009; Zhang and Li, 2014). The finding that the gut microbiota might be involved in the pathogenetic process of IBD sheds light on a promising and novel method involving the targeting of the gut microbiota to protect against IBD. In recent years, a number of microbiota-based strategies, such as probiotics and fecal microbiota transplantation, have been found to be effective in IBD treatment, but increasing the efficacy of these approaches is difficult (Ni et al., 2017). Therefore, novel protective methods might combine the advantages of immune modulators and the gut microbiota to prevent IBD-induced deleterious inflammation.

Interestingly, parasite or parasite-derived soluble antigens reportedly have the capacity to modulate host immunity (Maizels et al., 2018). For instance, *Tritrichomonas muris* induces type 2 immunity in the gut (Howitt et al., 2016); *Heligmosomoides polygyrus* suppresses the IL-33 pathway (Zaiss et al., 2013); *Schistosoma mansoni* soluble egg antigen promotes T helper 2 (Th2) polarization (Kaisar et al., 2018); and *S. mansoni* cercariae infection induces a predominantly immune-modulated response, which might suppress the proinflammatory Th1 response induced by DSS treatment (Winkel et al., 2018). In addition, parasites might shift the host’s immune state by modifying the microbiota and then altering healthy conditions (Eleftheriou, 2020). Given the dual effects of parasites on immunity and the microbiota, taking advantage of parasite infection could be a promising strategy for protecting against IBD. In reality, increasing lines of evidence show that the incidence of IBD is negatively correlated with the parasite exposure level (Geremia and Arancibia-Carcamo, 2017), and the applications of parasite infection to cure immune-related diseases, including asthma and IBD, have achieved some positive results in preclinical trials (Sann et al., 2013; Stiemsma et al., 2015; Wang M. et al., 2017). However, all these therapies (therapeutic strategies based on parasite-related immune modulation) are inevitably associated with deleterious effects caused by parasite infection. To minimize the detrimental influence of parasites, we explored the use of optimal parasites. Because hepatic granuloma and fibrosis formation depend on the immunogenic substances of *Schistosoma japonicum* (Sj) eggs (He et al., 2020), mSjci does not evoke granulomatous and fibrotic responses other than mild cercarial dermatitis. Thus, mSjci was considered as a candidate approach for protecting against IBD.

In the present study, we infected DSS-induced colitis mice with mSjci and assessed the protective effect through the observation of clinical signs, such as the body weight, diarrhea, total colon length, and histological configuration. Furthermore, we investigated the role of mSjci in the immune response by detecting the levels of IL-10^+^ cells, IFN-γ^+^ cells and regulatory T (Treg) cells by flow cytometry and its effects on the intestinal microbiota by 16S rRNA sequencing. Our findings revealed a new method that fuses the advantages of anti-inflammatory activity and microbiota modulation to achieve protection against IBD-induced detrimental inflammation.

## Materials and Methods

### Animals

Six-week-old male BALB/c mice (each weighing 18–20 g) were purchased from the Beijing Vital River Laboratory Animal Center (Beijing, China) and housed in a specific pathogen-free (SPF) environment with appropriate temperature and humidity conditions and a 12-h dark/light cycle. All the mice were given free access to sterilized water and standard-compliant food. Efforts were made to minimize the number of mice used in the experiments and their suffering. All animal experiments were approved by the Medical Research Ethics Committee of Sun Yat-sen University and conformed to the Chinese National Institute of Health Guide for the Care and Use of Laboratory Animals (No. 2017-008).

### Monosexual Sj Cercariae Infection

Positive *Oncomelania hupehensis* snails containing monosexual *S. japonicum* cercariae (mSjc) were obtained from the National Institute of Parasitic Diseases, Chinese Center for Disease Control and Prevention in Shanghai. These *O. hupehensis* snails were exposed to single *S. japonicum* miracidia, and 6 weeks later, cercariae were harvested as reported previously (Sombetzki et al., 2018). To ensure mSjc infection, each mouse was infected with the indicated number of either male or female Sj cercariae from a single *O. hupehensis* snail as previously described (Kaplan, 2015). The positive snails were illuminated for 2–4 h to induce cercariae shedding. Afterward, the cercariae were counted under a stereoscope and gathered in 200 μl of saline on a coverslip. The mice were then depilated on the abdomen and infected percutaneously by placing the coverslip containing mSjc on the naked skin of the mice for 30 min.

### DSS-Induced Colitis and Treatment

**FIGURE 1:**
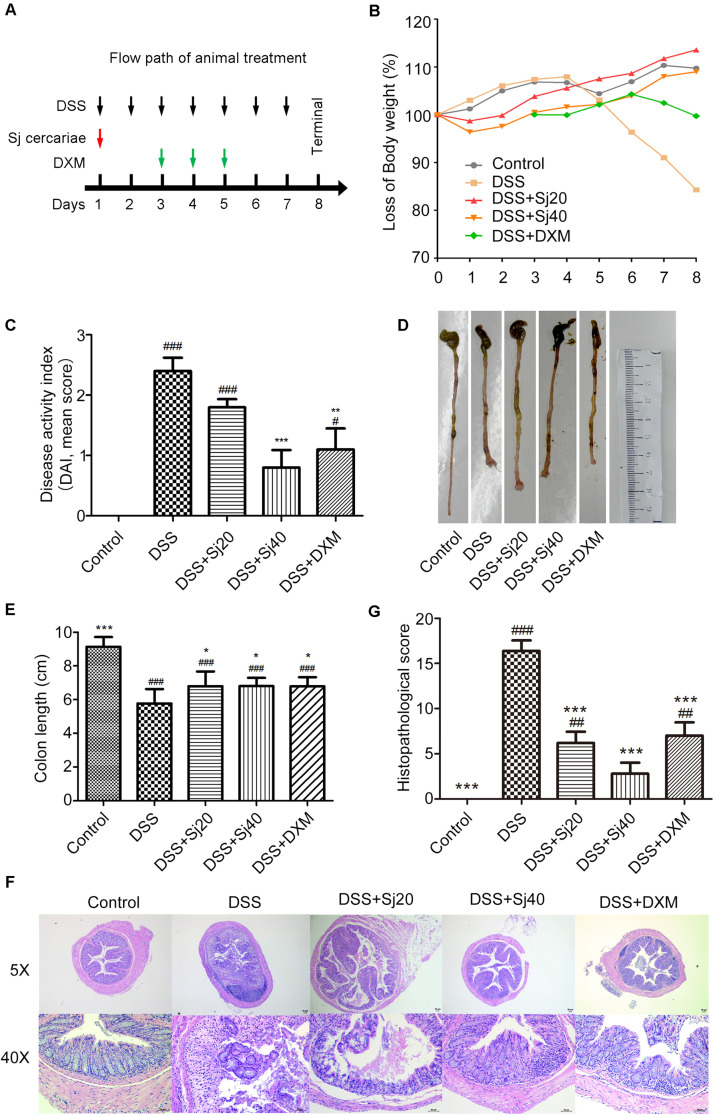
Treatment with mSjci attenuates the clinical scores of mice induced by DSS treatment. **(A)** Diagram showing the entire animal treatment process. **(B)** The body weight loss of mice in the five indicated groups was monitored for 7 consecutive days. **(C)** The disease activity index (DAI) of each group was evaluated on the 7th day of the experiment. The data are presented as the means ± standard deviations (SDs; *n* = 5). **(D,E)** The whole colon of each mouse was dissected for photography **(D)**, and the colon length was measured **(E)**. The data (means ± SDs; *n* = 5) were analyzed by one-way ANOVA. **(F)** Representative haematoxylin and eosin (H&E) staining showed abscesses, villus blunting, intestinal mucosa disruption, muscle layer thickening, epithelial ulcerations, goblet cell disruption and inflammatory infiltration. **(G)** The histopathological scores of colonic sections were calculated and compared between groups **(E)**. The data (means ± SDs; *n* = 5) were analyzed by one-way ANOVA. ^#^*p* < 0.05, ^##^*p* < 0.01, ^###^*p* < 0.001 compared with the control group; *p < 0.05, **p < 0.01, ***p < 0.001 compared with the DSS group.

All the mice were randomly divided into the following five groups (10 mice in each group): DSS group, DSS + Sj20 group, DSS + Sj40 group, DSS + DXM group and control group. The entire animal treatment procedure lasted 7 consecutive days, as displayed in the flow diagram (Figure 1A). First, all the mice administered DSS (DSS, DSS + Sj20, DSS + Sj40, and DSS + DXM groups) were intragastrically fed a solution of 2.5% (w/v) DSS (MP Biochemical, Santa Ana, CA, United States) for 7 consecutive days to induce acute colitis according to a previously described method (Abraham and Cho, 2009; Khan et al., 2019). The mice in the DSS + Sj20 and DSS + Sj40 groups were percutaneously infected with 20 and 40 mSjc, respectively, after the first DSS administration. The mice in the DSS + DXM group were intraperitoneally injected with dexamethasone (DXM) at a dose of 1 mg/kg/day from days 3 to 8 after DSS treatment as reported previously (Wang L. et al., 2017). The mice in the control group were intraperitoneally injected with the same volume of saline as that administered to the DSS + DXM group. The weight loss, rectal bleeding and diarrhea were monitored daily for 7 sequential days to assess the disease activity index (DAI) (referring to Table 1) as described previously (Wang L. et al., 2017). The mice were euthanized 8 days after the first DSS administration, the serum was collected, and the colons and spleens were dissected. After measuring the total colon lengths, the colons were divided into two parts. The distal segment of each colon was sampled for histological morphology examination, and the remainder was preserved for myeloperoxidase (MPO) and *N*-acetylglucosaminidase (NAG) activity measurements.

**TABLE 1:** Assessment of disease activity index (DAI).

**Body weight loss**	**Stool**	**Bleeding**	**Score**
<2%	Normal	No rectal bleeding	0
2–5%	Softer stool	Weak hemoccult	1
5–10%	Moderate diarrhea	Visual blood in stool	2
10–15%	Diarrhea	Fresh rectal bleeding	3
≥15%	–	–	4

### Histopathological Analysis

The distal segment of mouse colon was dissected, fixed in 4% paraformaldehyde for 24 h and embedded in paraffin according to previously described procedures (Zhou et al., 2019). Afterward, a 5-μm-thick colon tissue section was prepared with a histocryotome and then stained with haematoxylin and eosin (H&E). The histopathological score was evaluated based on the degree of inflammation and histopathological injury according to a previously described method (Wang L. et al., 2017).

The seven indexes used for histopathological score calculations included the extent of inflammation, infiltration of neutrophils and lymphocytes, extent of crypt damage, crypt abscesses, submucosal oedema, loss of goblet cells and reactive epithelial hyperplasia (referring to Table 2).

**TABLE 2:** Histopathological scores.

**Grade**	**Extent of inflammation**	**INL^a^**	**Extent of crypt damage**	**Crypt abscesses**	**Sub-mucosal oedema**	**Loss of goblet cells**	**REH^a^**
0	None	None	None	None	None	None	None
1	Mucosa	Focal	Basal one third	Focal	Focal	Focal	Focal
2	Mucosa to submucosa	Multifocal	Basal two thirds	Multifocal	Multifocal	Multifocal	Multifocal
3	Mucosa to muscle layer	Diffuse	Entire crypt damage	–	Diffuse	Diffuse	Diffuse
4	Transmural	–	Crypt damage + ulceration	–	–	–	–

*^*a*^INL, infiltration neutrophils + lympho-histiocytes; REH, reactive epithelial hyperplasia.*

### Assay of Epithelial Barrier Permeability

The intestinal epithelial barrier permeability was tested using a permeability tracer (FITC-labeled dextran) as described previously (Di Sabatino et al., 2012). Briefly, the mice were maintained fasted and deprived of water overnight, and FITC-labeled dextran (Sigma-Aldrich, Saint Louis, MO, United States) was then administered to each mouse intragastrically at a dose of 44 mg/100 g body weight. Four hours later, the mouse blood was drawn, and serum was reserved for measurement of the fluorescence intensity, which was obtained by reading the absorbance at 525 nm with a Spark multimode microplate reader (TECAN, Switzerland). The fluorescence intensity of each sample was then calculated according to a standard curve ranging from 2,000 to 100 μg/ml (Zhang and Li, 2014).

### Measurement of Myeloperoxidase (MPO) and *N*-Acetylglucosaminidase (NAG) Activities

The infiltration of inflammation-related cells in the mouse colon was determined by measuring the MPO (indicator of neutrophil infiltration) and NAG (indicator of macrophage infiltration) activities in the homogenized colon tissues using an MPO ELISA kit (#69-28146, MSKBIO, Wuhan, China) and a NAG ELISA kit (#59-45262, MSKBIO, Wuhan, China) according to the manufacturer’s recommended protocols. The results were presented as absolute values of MPO and NAG activities based on the optical density per gram of tissue.

### Flow Cytometry

The mouse spleens were homogenized thoroughly on ice and centrifuged at 1,500 rpm for 5 min to prepare a single-cell suspension. The red blood cells in the cell suspension were then removed using red blood cell lysis buffer (Boster Bio, CA, United States) according to the manufacturer’s instructions. Subsequently, the Fc receptors on the cell surfaces were blocked on ice for 30 min. Surface and intracellular staining were performed to determine the percentages of Treg cells and INF-γ- or IL-10-producing cells. FITC-labeled anti-CD4 (BD Biosciences, NJ, United States) and PE-labeled anti-CD25 (BD Biosciences) antibodies were utilized for surface staining. For intracellular staining, the cells were fixed and permeabilized using fixation/permeabilization buffer (BD Biosciences), washed three times and then incubated with APC-conjugated anti-IL-10 (BD Biosciences) and APC-conjugated anti-INF-γ (BD Biosciences) antibodies. All gating was based on the use of respective isotype control antibodies, and the analysis was performed using FlowJo software (Tree Star, Inc., Ashland, OR, United States) (Gkouskou et al., 2014).

### Gut Microbiota Profiling

A total of 25 mouse fecal samples from the indicated groups (5 mice from each group) were collected before sacrifice. Total genomic DNA was extracted using a DNA Extraction Kit (QIAGEN, Hilden, Germany) following the manufacturer’s protocol. The DNA quality and quantity were tested with a NanoDrop instrument and by agarose gel electrophoresis. The extracted DNA was diluted to a concentration of 1 ng/μl and stored at −20°C until further processing. The diluted DNA was used as the template for the PCR amplification of bacterial 16S rRNA genes utilizing barcoded primers and Takara Ex Taq (TaKaRa, Dalian, China). The V3-V4 variable regions of 16S rRNA genes were amplified with the universal primers 343F and 798R. The amplicon quality was visualized by gel electrophoresis, and the amplicons were purified with AMPure XP beads (Beckman Coulter, Brea, CA, United States) and amplified for another round of PCR. After re-purification with the AMPure XP beads, the final amplicons were quantified using a Qubit dsDNA assay kit. Equal amounts of purified amplicons were pooled for subsequent sequencing. Clean reads were subjected to primer sequence removal and clustering to generate operational taxonomic units (OTUs) with a 97% similarity cutoff using Vsearch software (Goto et al., 2015). The representative read of each OTU was selected using the QIIME package. All representative reads were annotated and blasted against Greengenes (16S rDNA) using the RDP classifier (with a confidence threshold of 70%) (Ni et al., 2017).

### Statistical Analyses

All the data are presented as the means ± standard deviations (SDs). The significance of the differences between two groups was tested by two-tailed Student’s *t*-test, whereas one-way ANOVA was adopted for the comparisons of more than two groups. In all cases, a *p-*value less than 0.05 was considered to indicate significance. All the analyses were conducted using GraphPad Prism software (version 7.0, San Diego, CA, United States).

## Results

### Mice Treated With mSjci Show Attenuated Clinical Signs of DSS-Induced Colitis

To gain insights into the impact of mSjci on colitis, colitis was induced in mice through the administration of DSS and the mice were then administered several treatments, namely, infection with mSjc and intraperitoneal injection of DXM (positive control). The treatment procedure lasted 7 consecutive days, as displayed in the flow path (Figure 1A). The body weight of the mice in each group was recorded over a 7-day period, and the data showed that DSS-treated mice started to show a sharp loss of body weight starting 5 days post treatment, and as expected, the DSS + DXM treatment significantly ameliorated this weight loss. In contrast, the mice in the control group gradually gained body weight. Surprisingly, we found that the DSS + mSjci-treated mice exhibited improved body weight gain than the mice in the DSS and DSS + DXM groups (Figure 1B). Moreover, the DAI of mice was assessed on the 7th day after treatment through the use of scoring systems based on weight loss, diarrhea and bleeding (see section “Materials and Methods”). The results revealed that DSS induced severe clinical colitis manifestations, whereas treatment with DSS + Sj40 and DSS + DXM obviously reversed these detrimental effects (Figure 1C, *p* < 0.001 and *p* < 0.01, respectively). Furthermore, the DSS + mSjci treatment attenuated/reduced the symptoms of colitis in a dose-dependent manner (Figure 1C).

In addition, the mouse colon was dissected on the 8th day. Macroscopic images were captured at the same scale, and the colon length was then calculated. As expected, the mice treated with DSS exhibited a conspicuously shortened colon compared with the those in the normal control group. Of note, the colon of DSS-treated mice administered mSjci or DXM was not equal to that of normal mice but was relatively longer than that of the mice in the DSS group, as shown by the macroscopic observations (Figure 1D) and quantification analyses (Figure 1E, both *p* < 0.05). The histological configuration of the mouse colon was then investigated, and we found that DSS treatment led to typical pathological features of colitis, including abscesses, epithelial ulcerations, villus blunting, intestinal mucosa disruption, muscle layer thickening, goblet cell disruption, and infiltration by a multitude of inflammatory cells (Figures 1F,G), compared with the control mice. In contrast, the mice treated with DSS + Sj20, DSS + Sj40, and DSS + DXM showed less damage to the colon structure than those treated with DSS alone (Figures 1F,G, both *p* < 0.001). Overall, these data suggest that mice treated with mSjci showed attenuated clinical signs/lesions of DSS-induced colitis.

### Mice With DSS-Induced Colitis Exhibit Less Impaired Intestinal Permeability After Treatment With mSjci

**FIGURE 2:**
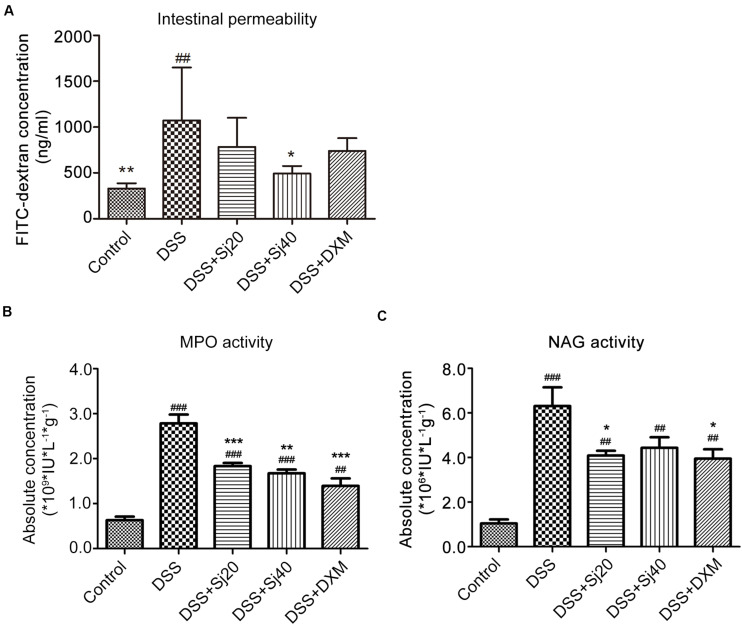
The impairment of the intestinal permeability induced by IBD is decreased after treatment with mSjci. **(A)** The FITC-dextran concentration (reflecting intestinal permeability) was calculated according to a standard curve. The data (means ± SDs; *n* = 5) were analyzed by one-way ANOVA. **(B)** The mice were treated as indicated, and 7 days later, the myeloperoxidase (MPO) and **(C)** *N*-acetylglucosaminidase (NAG) activities in colon tissue were determined by ELISA. The bars represent the means ± SD (*n* = 5). The significant differences among the groups were analyzed by one-way ANOVA. ^##^*p* < 0.01, ^###^*p* < 0.001 compared with the control group; *p < 0.05, **p < 0.01, ***p < 0.001 compared with the DSS group.

We performed an epithelial barrier permeability assay. The serum of mice intragastrically administered FITC-dextran was subjected to FITC fluorescence intensity measurement. As expected, DSS clearly elevated the FITC intensity by more than twofold compared with that of the control group, and this finding indicated that the structure of the intestinal villus was markedly impaired by DSS, which leads to enhanced intestinal permeability (Figure 2A, *p* < 0.01). In contrast, the treatment of mice with DSS-induced colitis with 40 mSjc decreased the fluorescence intensity to an approximately normal level (Figure 2A, *p* < 0.05), which suggested a less impaired intestinal structure.

We further determined the extravasation of neutrophils and macrophages in the mouse colon by determining the activities of MPO (indicator of infiltrated neutrophils) and NAG (indicator of infiltrated macrophages), respectively. To this end, homogenized mouse colon tissues were subjected to ELISA. The results showed that DSS markedly promoted increases in the activities of both MPO and NAG (Figures 2B,C, both *p* < 0.001). However, the DSS + Sj20 and DSS + Sj40 treatments visibly decreased the activity of MPO by 20% (Figure 2B, *p* < 0.001) and 34% (Figure 2B, *p* < 0.001), respectively. Nevertheless, a considerable reduction in NAG activity was observed only in the 20 mSjc-treated mice (Figure 2C, *p* < 0.001). Notably, no obvious difference was found among the DSS + Sj20, DSS + Sj40, and DSS + DXM groups (Figure 2C, *p* > 0.05). Overall, treatment with mSjci decreased the impairment of the intestinal permeability of the colons of mice with DSS-induced colitis.

### Mice With DSS-Induced Colitis Display a Mitigated Proinflammatory Context in the Spleen After Treatment With mSjci

**FIGURE 3:**
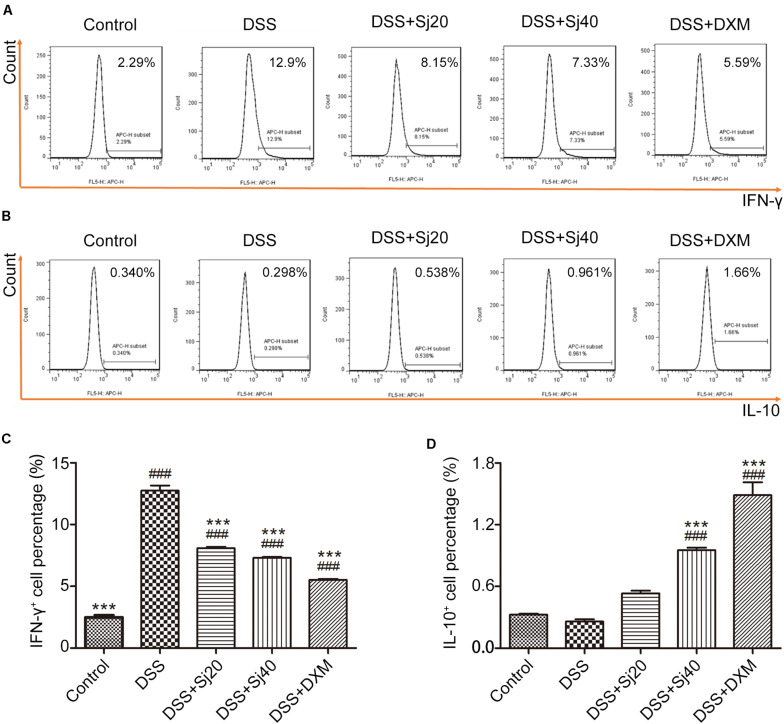
Mice treated with mSjci display decreased production of the proinflammatory cytokine IFN-γ and enhanced expression of the anti-inflammatory cytokine IL-10. **(A)** At the termination of the animal experiment, the spleen of each mouse was eviscerated and then analyzed by flow cytometry to test the production of IFN-γ. **(B)** Similarly, the expression of IL-10 was determined by flow cytometry to analyze the percentage of IL-10^+^ cells. **(C,D)** Quantification of the cells positive for IFN-γ **(C)** or IL-10 **(D)** in each group. The data (means ± SDs; *n* = 5) were analyzed by one-way ANOVA. ^###^*p* < 0.001 compared with the control group; ***p < 0.001 compared with the DSS group.

IBD is characterized by excess production of proinflammatory cytokines, such as IFN-γ, and downregulation of anti-inflammatory cytokines, such as IL-10. The levels of these cytokines commonly suggest the disease activity of IBD. We thus detected the levels of IFN-γ and IL-10 in the spleens of mice with DSS-induced colitis after treatment with mSjci by flow cytometry. Mice exposed to DSS displayed a sixfold higher percentage of IFN-γ^+^ cells (Figures 3A,C, *p* < 0.001) than the control mice. However, the DSS + Sj20, DSS + Sj40, and DSS + DXM treatments reduced this percentage to a relatively lower level than that obtained with DSS treatment alone (Figures 3A,C, both *p* < 0.001). Conversely, DSS induced a decrease in the percentages of IL-10^+^ cells in the mouse spleen, whereas the DSS + Sj20, DSS + Sj40, and DSS + DXM treatments markedly elevated the proportion of IL-10^+^ cells, which suggested that mSjci exerted an anti-inflammatory effect (Figures 3B,D). These results demonstrated that mSjci treatment induced mitigation of the proinflammatory context in the spleens of mice with DSS-induced colitis by enhancing IL-10 production and restraining IFN-γ expression.

### Mice With DSS-Induced Colitis Show an Enhanced CD4^+^CD25^+^ Treg Cell Population in the Spleen After Treatment With mSjci

**FIGURE 4:**
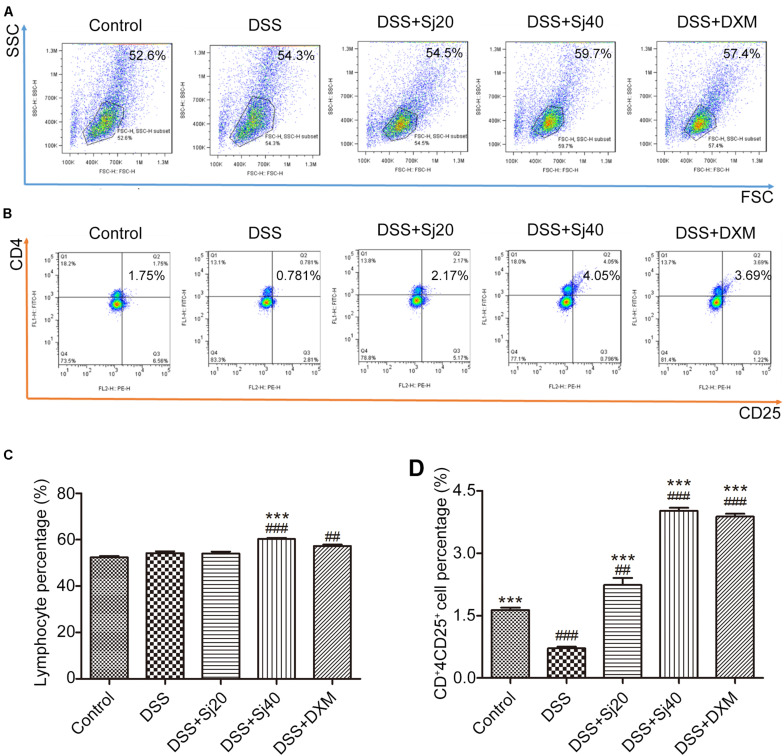
Mice treated with mSjci show elevated populations of lymphocytes and the CD4^+^CD25^+^ Treg cell subset. **(A,B)** The percentages of lymphocytes **(A)** and CD4^+^CD25^+^ Treg cells **(B)** was determined by flow cytometry. **(C,D)** The percentages of lymphocytes **(C)** and Treg cells **(D)** were quantified by flow cytometry analysis. The data (means ± SDs; *n* = 5 in triplicate) were analyzed by one-way ANOVA. ^##^*p* < 0.01, ^###^*p* < 0.001 compared with the control group; ***p < 0.001 compared with the DSS group.

Because mSjci exerted immunosuppressive effects by affecting the production of cytokines (IL-10 and IFN-γ) that are generally regulated by immune cells, including lymphocytes such as Treg cells (Dobrzanski et al., 2012), we tested the percentage of total lymphocytes and CD4^+^CD25^+^ Treg cells in the mouse spleens to assess whether mSjci altered the expression of IL-10 and IFN-γ by governing the Treg cell percentage. For this purpose, we performed flow cytometry using antibodies against CD4 and CD25 (specific marker of Treg cells) and found that DSS + Sj40 evidently boosted the percentage of lymphocytes compared with that found with DSS (Figures 4A,C, *p* < 0.001). Further analyses of the Treg cell ratio indicated that the DSS + Sj20, DSS + Sj40, and DSS + DXM treatments led to repression of the Treg cell population induced by DSS (Figures 4B,D, both *p* < 0.001) and evoked a higher percentage of Treg cells than the control treatment (Figures 4B,D). The above-described results suggested that the mSjci treatment might regulate the expression of IL-10 and IFN-γ in mice with DSS-induced colitis by driving expansion of the Treg cell population in the spleen.

### The Gut Structure of Mice Display a Modulated Community Structure After Treatment With mSjci

In the present study, to explore whether the protective effect of mSjci on IBD resulted from modulation of the gut microbiota, we performed 16S rRNA gene sequencing as described in the Section “Materials and Methods.” In total, 710,488 valid reads were obtained from 25 fresh stool samples from the five indicated groups of mice, and an average of 28,419 counts were obtained for each sample (Supplementary Figure 1). The raw data were then filtered at a 97% similarity level, which resulted in the generation of 1,402 OTUs (Supplementary Table 1), and 46 of these OTUs were shared by all 25 samples (Supplementary Figure 2). The most frequently shared OTUs included Coprococcus_1, Streptococcaceae and Bacteroidales_S24_7_group (Supplementary Table 2).

**FIGURE 5:**
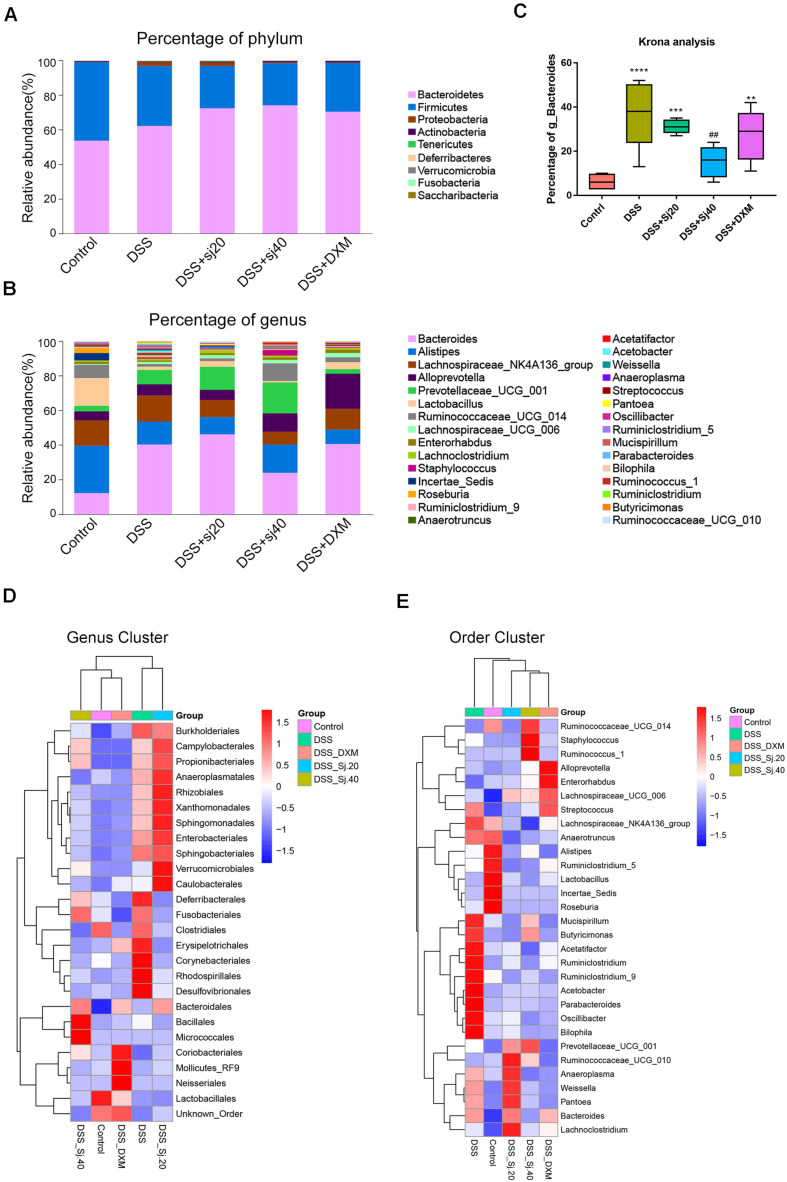
The community structure of the gut microbiota of mice is modulated by treatment with mSjci. **(A,B)** Relative abundances of the top 30 most important gut microbiota constituents at the phylum level **(A)** and genus level **(B)** in each group, these values were derived from the 16S rRNA sequencing of fresh stool samples. **(C)** A Krona analysis revealed the difference in the Bacteroides abundance among the groups. The data (means ± SDs; *n* = 5) were analyzed by one-way ANOVA. **(D,E)** A cluster analysis displayed the different abundances of bacterial genera **(D)** and orders **(E)** among the groups. ^##^*p* < 0.01 compared with the control group; **p < 0.01, ***p < 0.001, ****p < 0.0001 compared with the DSS group.

Afterward, we analyzed the community structure of the gut microbiota in all five groups at the phylum (Figure 5A) and genus levels (Figure 5B) by calculating the percentage of phyla and genera and then performing a statistical analysis. The results indicated that the phyla Bacteroidetes and Firmicutes were the most abundant in all five groups, but no significant trend coinciding with the protective effect of mSjci was found (Figure 5A). We thus further investigated the alterations at the genus level among the five groups, and two-way ANOVA was used to evaluate the significant differences. Surprisingly, we observed that DSS induced an obvious increase in the Bacteroides percentage compared with that found in the control group (Figure 5B, *p* < 0.001), and the percentage of Bacteroides was decreased after treatment with DSS + Sj40 (Figure 5B, *p* < 0.001) compared with the Bacteroides percentage in the control mice, which suggesting that Bacteroides might serve as a deleterious factor during the progression of IBD. Then, a Krona analysis was further performed to assess the difference in the Bacteroides percentage among the groups, and the results showed that g_Bacteroides was notably enriched by DSS exposure (Figure 5C, *p* < 0.001) but depleted in the DSS + Sj20- and DSS + DXM-treated mice (Figure 5C, both *p* < 0.01). Surprisingly, the treatment of DSS-treated mice with Sj40 returned the level of g_Bacteroides to nearly the normal levels detected in the control group (Figure 5C, *p* > 0.05). Moreover, a cluster analysis revealed visible differences in the bacterial genera (Figure 5D) and orders (Figure 5E) among the groups, and the mice with improved IBD symptoms exhibited, at least partly, a reversed bacterial community structure that showed similarity to that of the normal control mice. These results suggest that mSjci might exert protective effects on DSS-induced IBD by modulating the overall community structure of the gut microbiota.

### The Diversity of the Gut Microbiota of DSS-Treated Mice Is Elevated by mSjci Treatment

**FIGURE 6:**
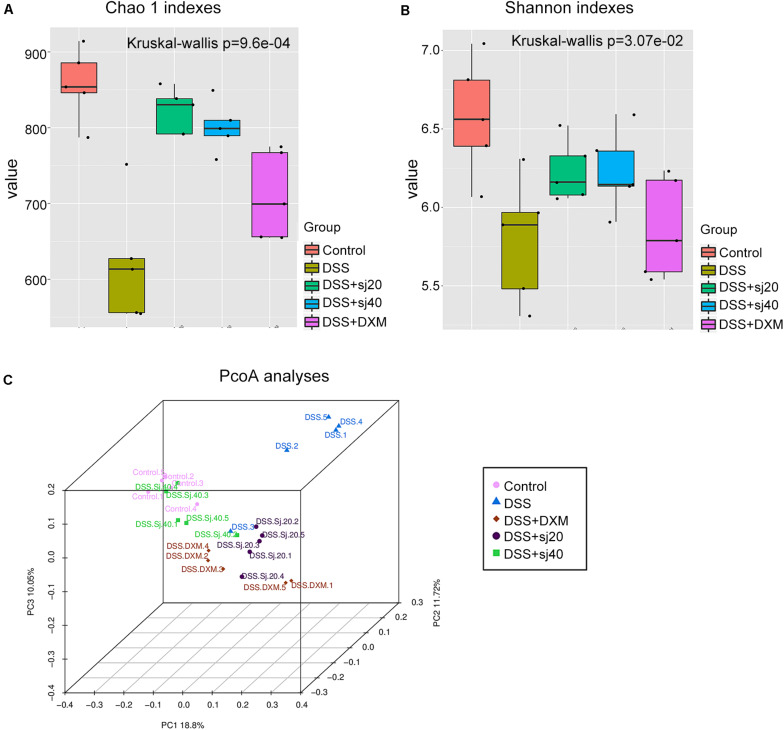
The diversity of the gut microbiota of mice with IBD is elevated by treatment with mSjci. **(A)** The richness of the taxa was analyzed with the Chao 1 index. **(B)** The number of different taxa and their abundance were assessed using the Shannon index. The error bars represent the median and 25th–75th percentiles of the alpha diversity metrics obtained for each group. **(C)** The distance among the samples from each group of mice was visualized by 3D principal coordinate analysis (PCoA) score plots (each point represents one sample).

Although we observed that IBD could elevate the g_Bacteroides percentage, as described above (Figure 5C), whether the diversity of the gut microbiota altered by exposure to DSS was associated with mSjci treatment remained unknown. To answer this question, we analyzed alpha and beta diversities of the gut microbiota among the groups. For the alpha diversity analysis, six statistical methods were applied (Supplementary Table 3), and among these, the Chao 1 index (Figure 6A), Shannon index (Figure 6B) and observed species (Supplementary Figure 3) displayed the most significant and consistent tendency with respect to the gut microbiota richness in the five groups. All these results indicated that the mice with DSS-induced IBD exhibited a dramatic decrease in bacterial richness, whereas the DSS + Sj20, DSS + Sj40, and DSS + DXM treatments increased the diversity of bacteria. Notably, the mice treated with mSjci presented a higher bacterial diversity than the DSS + DXM-treated mice, which suggested that the DSS + mSjci treatment might exert better protective effects on IBD than the DSS + DXM treatment. For assessment of the beta diversity, we performed a beta diversity index analysis (Supplementary Figure 4, showing the distance of samples between groups) and a principal coordinate analysis (PCoA) (Figure 6C, showing the difference of samples between groups). The beta diversity index analysis revealed that the DSS + Sj40 and control groups were clustered closely, the DSS + Sj20 and DSS + DXM groups were clustered close to each other, and the DSS group was relatively isolated, which implied that the DSS + Sj40 treatment exerted a better protective effect on IBD than the DSS + DXM and DSS + Sj20 treatments (Supplementary Figure 4). The PCoA showed that the DSS group was distal to the other groups on a 3D scale (Figure 6C). Of note, the DSS + Sj40 group was located close to the control group (Figure 6C), which was in line with the results of the beta diversity index analysis. These results suggest that mSjci might protect against DSS-induced IBD by modulating the depressed diversity of the gut microbiota.

### MSjci Governs the Abundance of Specific Bacterial Families and Genera

**FIGURE 7:**
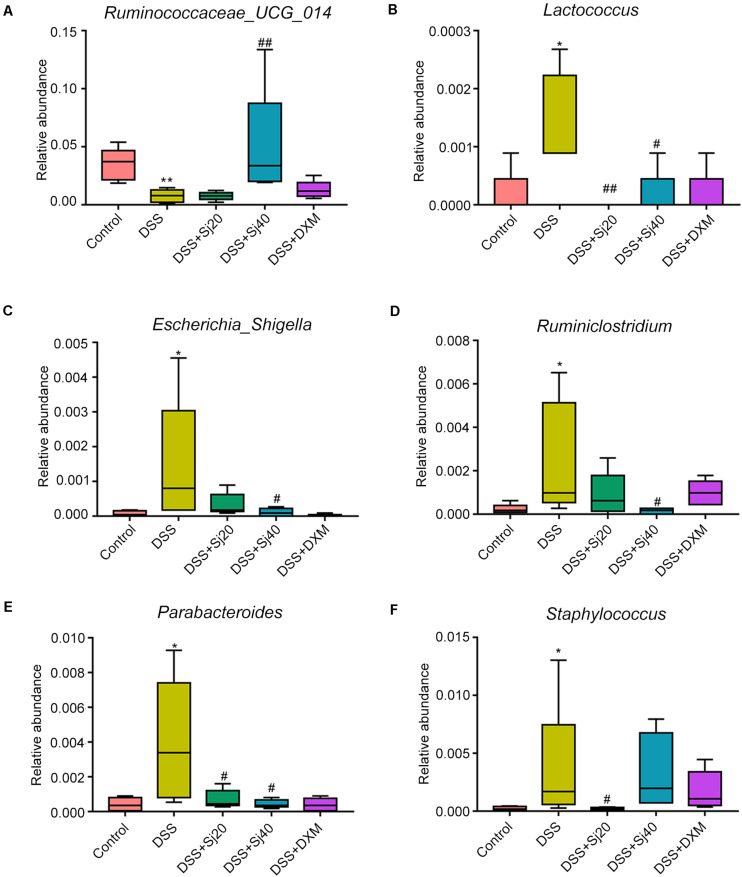
mSjci governs the abundance of specific bacterial genera. **(A–F)** DSS-induced IBD strikingly reduced the abundance of *Ruminococcaceae_UCG_014* **(A)** but enhanced the abundances of *Lactococcus* **(B)**, *Escherichia_Shigella* **(C)**, *Ruminiclostridium* **(D)**, *Parabacteroides* **(E)**, and *Staphylococcus* **(F)**, whereas mSjci and DXM protected against this perturbation of the genera. ^#^*p* < 0.05, ^##^*p* < 0.01 compared with the DSS group; *p < 0.05, **p < 0.01 compared with the control group.

**FIGURE 8:**
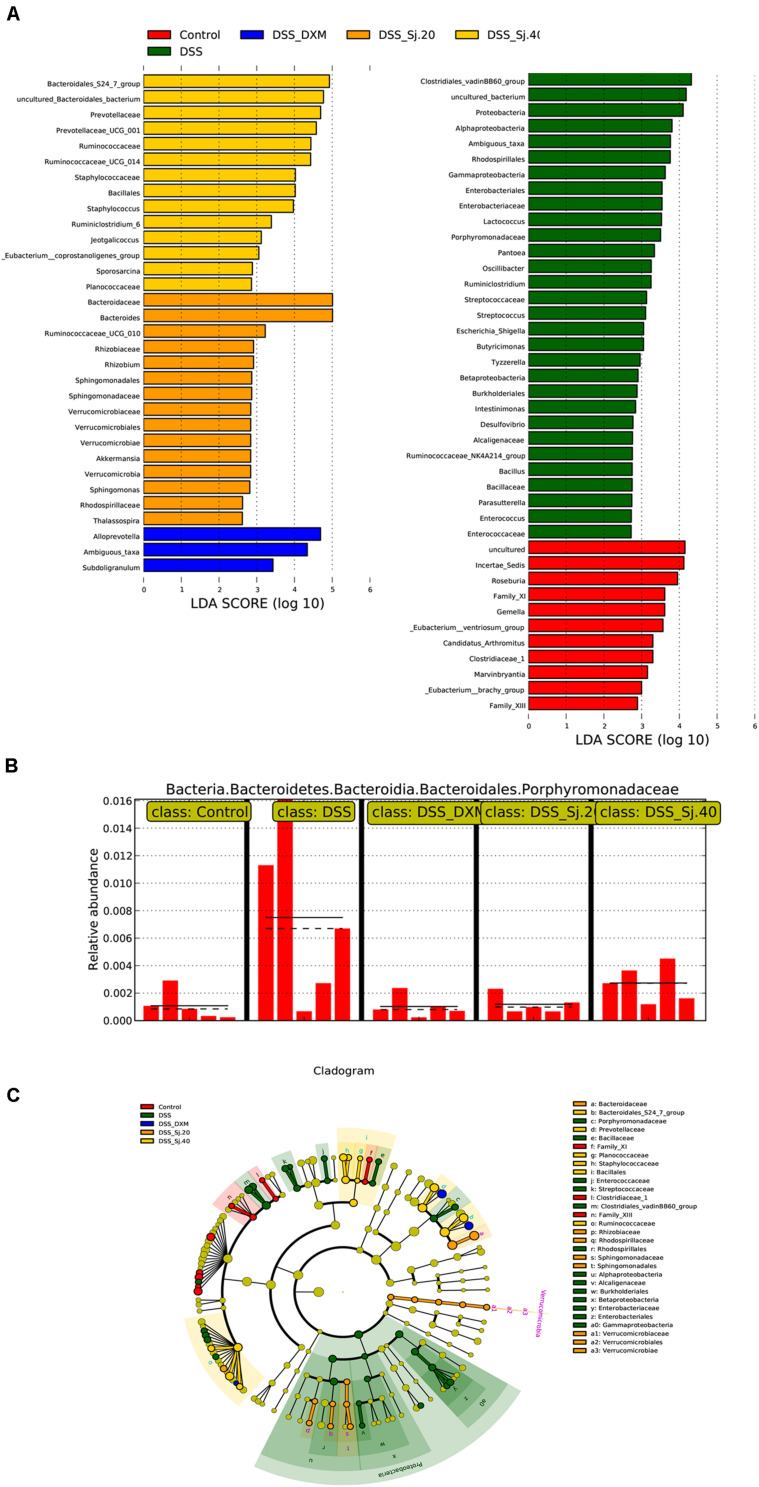
mSjci governs the abundance of taxa and specific bacterial families. **(A)** The most differentially abundant taxa were identified through the LDA (linear discriminant analysis) score generated using the LEfSe method. **(B)** The relative abundance of the bacterial family showed marked differences between the IBD and treatment groups. **(C)** Cladogram displaying the taxa showing differences among the five groups. The colored regions represent individual groups. The colored nodes represent the microbial groups that might play a critical role in the corresponding groups.

To further elucidate the mechanism underlying the effect of mSjci on IBD, we continued to explore the specific bacterial family or genus that showed stable changes during the IBD process. Hence, we subsequently conducted multivariate analyses using ANOVA, Kruskal–Wallis and linear discriminant analysis (LDA) coupled with effect size measurement (LEfSe) methods. The ANOVA differential analysis revealed that IBD distinctively altered the abundances of some bacterial genera, including increases in Tyzzerella, Oscillibacter, and Bilophila and decreases in Alistipes and Subdoligranulum compared with the abundances in the DSS + mSjci and DSS + DXM groups (Supplementary Figure 5). Further Kruskal–Wallis analysis showed that at the genus level, IBD strikingly reduced the quantity of *Ruminococcaceae_UCG_014* (Figure 7A) but enhanced the abundances of *Lactococcus* (Figure 7B), *Escherichia_Shigella* (Figure 7C), *Ruminiclostridium* (Figure 7D), *Parabacteroides* (Figure 7E), and *Staphylococcus* (Figure 7F). Based on the LEfSe results (Figure 8A), we found that *Lactococcus, Escherichia_Shigella*, and *Ruminiclostridium* were among the most abundant genera after the establishment of IBD. Of note, *Escherichia_Shigella* was found to exhibit the best association with the change in the IBD status. Therefore, mSjci likely functions in IBD by governing the richness of *Escherichia_Shigella*. Moreover, at the family level, Porphyromonadaceae was markedly enriched at the active IBD state but dramatically depleted to the same level as that in the normal control group (Figure 8B), which indicated that Porphyromonadaceae might serve as the key bacterial family mediating IBD activity. Subsequently, a cladogram illustrated the most relevant clades among the five groups, which further supported the above-described results (Figure 8C). Collectively, these data indicate that mSjci might exert a protective effect on IBD by regulating the abundance of *Escherichia_Shigella*.

## Discussion

The present study provides evidence implying that mSjci might ameliorate DSS-induced colitis in mice. Treatment with mSjci obviously attenuated the symptoms of mice with DSS-induced colitis. Pathophysiologically, mSjci altered the balance between Th1 and Th2 cells by regulating the secretion of the cytokines IL-10 and IFN-γ and affecting the percentage of Treg cell subsets. In addition, mSjci reduced the inflammatory microbiota of mice with DSS-induced colitis compared with that of the control mice, which might account for the alteration in immune conditions caused by mSjci.

An increasing body of evidence shows that parasite therapies have achieved positive results for IBD (Gause et al., 2003; Ben-Ami Shor et al., 2013; Stiemsma et al., 2015; Wang M. et al., 2017). The administration of parasites, including *Hymenolepis diminuta*, *H. polygyrus*, *Trichuris suis*, and *Trichinella spiralis*, clearly ameliorate colitis by adjusting the immune response of the host, as reported previously (Wang M. et al., 2017). Of note, a randomized, placebo-controlled clinical trial of *T. suis* therapy showed promising and reliable effects on patients with active ulcerative colitis (Summers et al., 2005). Similarly, the present study showed that the decreased body weight (Figure 1B), shortened colon length (Figures 1D,E) and impaired epithelial barrier function (Figures 1F,G) in DSS-induced colitis were significantly improved by treatment with mSjci. Moreover, compared with the DSS group, the DAI (Figure 1C) was significantly decreased in the DSS + Sj40 group, the MPO (Figure 2B) activity levels were notably declined in the DSS + Sj20 and DSS + Sj40 groups, and the NAG (Figure 2C) activity levels were remarkably decreased in the DSS + Sj20 group, which suggested that mSjc infection could attenuate DSS-induced colitis activity in experimental mice.

Most parasites stimulate the host to induce Th2 cytokines, such as IL-10, while repressing Th1 cytokines, such as IFN-γ, according to the conventional Th1/Th2 balance theory (Ben-Ami Shor et al., 2013). After schistosome infection, an initial proinflammatory Th1 response is replaced by an anti-inflammatory Th2 response (Gause et al., 2003; Winkel et al., 2018). Based on this finding, we concurrently applied mSjci and DSS to mice and found that mSjci exerted protective effects on mice with DSS-induced colitis by suppressing proinflammatory Th1 cytokine (IFN-γ) production (Figures 3A,C) and promoting regulatory cytokine (IL-10) secretion (Figures 3B,D). However, the Th1/Th2 balance cannot fully explain how parasite therapy prevents this chronic inflammatory disease. The Treg cell subset reportedly plays a pivotal role in regulating innate immunity during chronic inflammatory diseases, such as IBD (Britton et al., 2019). Substantial evidence demonstrates that *Litomosoides sigmodontis* can protect against type 1 diabetes by inducing the Treg cell subset (Hubner et al., 2009). The increased percentage of Treg cells also participates in the modulation of the immune response and prevents the development of colitis in mice (Mo et al., 2007). Importantly, we discovered that the population of CD4^+^CD25^+^ Treg cells was significantly decreased in DSS-induced colitis but enhanced after treatment with mSjci. These findings coincide with previous reports showing that the Treg cell subset plays pivotal roles in controlling excessive immune responses and inducing immune tolerance (Mayne and Williams, 2013).

A multitude of studies have indicated that parasites and gut commensal bacteria play vital roles in the modulation of the immune response (Wang M. et al., 2017). An altered gut microbiota composition can lead to physiologically important changes in the intestinal environment (Weingarden and Vaughn, 2017). Furthermore, patients with IBD frequently show decreased microbiota diversity (Dicksved et al., 2008). In addition, *S. mansoni* infection reportedly limits the dysbiosis of the host’s intestinal microbiome and suppresses colitis (Floudas et al., 2019). In agreement with these findings, we observed repression of microbiota diversity and richness in mice with DSS-induced colitis, and these diversity and richness were increased after the administration of mSjci (Figures 6A,B), which suggested a shift from dysbiosis to eubiosis. As reported, the gut microbiota could contribute to IBD pathogenesis, and the process is tightly related to the Treg cell subset population (Britton et al., 2019), which implies that the upregulation of the Treg cell subset after mSjci treatment might result from the alteration of the gut microbiota. This finding provides new insights into the interaction between the microbiota and immune response, but further investigations are needed to determine their possible relationship.

The abundance of the gut microbiota might lead to differentially expressed cytokines (Pistollato et al., 2016). For example, *Oscillibacter*, which is enriched in mice with ulcerative colitis, is significantly positively correlated with the IL-6 and IL-1β levels (Wu et al., 2019), which implies that the gut microbiota might orchestrate immunity by regulating the secretion of cytokines. In this study, we found that the relative abundance of *Ruminococcaceae_UCG_014* was reduced in DSS-induced colitis but increased after mSjci infection (Figure 7A). Similar findings were obtained in a previous study, which showed that *Ruminococcaceae_UCG_014* is rebalanced after treatment with nitrate (McNabney and Henagan, 2017). Moreover, *Ruminococcaceae* can produce short-chain fatty acids, such as butyrate, which in turn facilitates colonic cancer cell apoptosis and reduces gut inflammation. *Parabacteroides*, which is enriched significantly during the ulcerative colitis exacerbation period compared with that during the remission period (Walujkar et al., 2018), serves as a key bacteria related to IBD (Huang et al., 2019). Moreover, we also found that *Lactococcus*, *Escherichia_Shigella*, *Ruminiclostridium*, and *Staphylococcus* were enriched in mice with IBD (Figures 7B–F), and these bacteria pass through the permeable intestine and cause worsened colitis. Notably, *Escherichia_Shigella* (at the genus level) exhibited the greatest enrichment at the active IBD state but was markedly depleted to the normal level after treatment with mSjci (Figure 7C), which indicated that mSjci might exert a protective effect on IBD mainly by regulating the abundance of *Escherichia_Shigella*. Mechanistically, parasites might reshape the community structure, diversity and richness of the gut microbiota by regulating the role of goblet cells and then promote the colonization of protective bacterial taxa (Dickson, 2016).

In summary, our findings provide evidence showing that mSjci could attenuate DSS-induced colitis in mice by inhibiting the production of IFN-γ, promoting the expression of IL-10 and enhancing the population of CD4^+^CD25^+^ Treg cells. Furthermore, the potential mechanism underlying the effect of mSjci on the immune response might rely on the repression of proinflammatory microbiota constituents. Our study provides a new approach for achieving protection against IBD-related detrimental inflammation and exhibits a broader application of mSjci to other immune-related diseases.

## Data Availability Statement

The raw data of the 16S rRNA sequencing for 25 fresh stools has been deposited in the NCBI Sequence Read Archive (SRA) under the project number PRJNA663139.

## Ethics Statement

The animal study was reviewed and approved by Animal Care and Use Committee of Sun Yat-sen University.

## Author Contributions

This project was designed and conceived by ZYL. ZYL and HLZ wrote the article. The experiments were performed by HLZ, XJZ, and DCS. ZC, WXC, and LWF analyzed the data and prepared the figures and tables. YL, PD, and WM participated in the study design and coordination. All authors read and approved the final manuscript.

## Conflict of Interest

The authors declare that the research was conducted in the absence of any commercial or financial relationships that could be construed as a potential conflict of interest.

## Supplementary Material

Supplementary Figure 1 The OTU level bar plot shows the summary of the total number of tags in the OTUs classified into each level (species, genus, family, order, class, phylum, and kingdom).

Supplementary Figure 2 The flower plot shows the unique and shared OTUs among all samples from the five groups.

Supplementary Figure 3 Alpha diversity analyses of the observed_species reveal the richness of the gut microbiota.

Supplementary Figure 4 Beta diversity analyses based on the hierarchical clustering method demonstrate the distance among the samples from the five groups.

Supplementary Figure 5 The abundance of some bacterial genera regulated by the DSS + DXM and DSS + mSjci treatment were revealed by an ANOVA of genera.

Supplementary Figure 6 The different genera between the four treatment groups and the DSS group show the significantly altered genera that are likely involved in the improvement of IBD.

Supplementary Table 1 OTU tables and taxonomic classifications of the 16S rRNA gene sequences.

Supplementary Table 2 Most abundant taxa and corresponding representative sequences derived from the OTU classification and annotation.

Supplementary Table 3 Alpha diversity analyses of OTU indexes show the differences in species abundance across all samples from the five groups.
