# Supplementary figures and images for: Monosexual Cercariae of Schistosoma japonicum Infection Protects Against DSS-Induced Colitis by Shifting the Th1/Th2 Balance and Modulating the Gut Microbiota
Source: Front Microbiol. 2021 Jan 5;11:606605. doi: 10.3389/fmicb.2020.606605 (PMC7813680; doi:10.3389/fmicb.2020.606605)

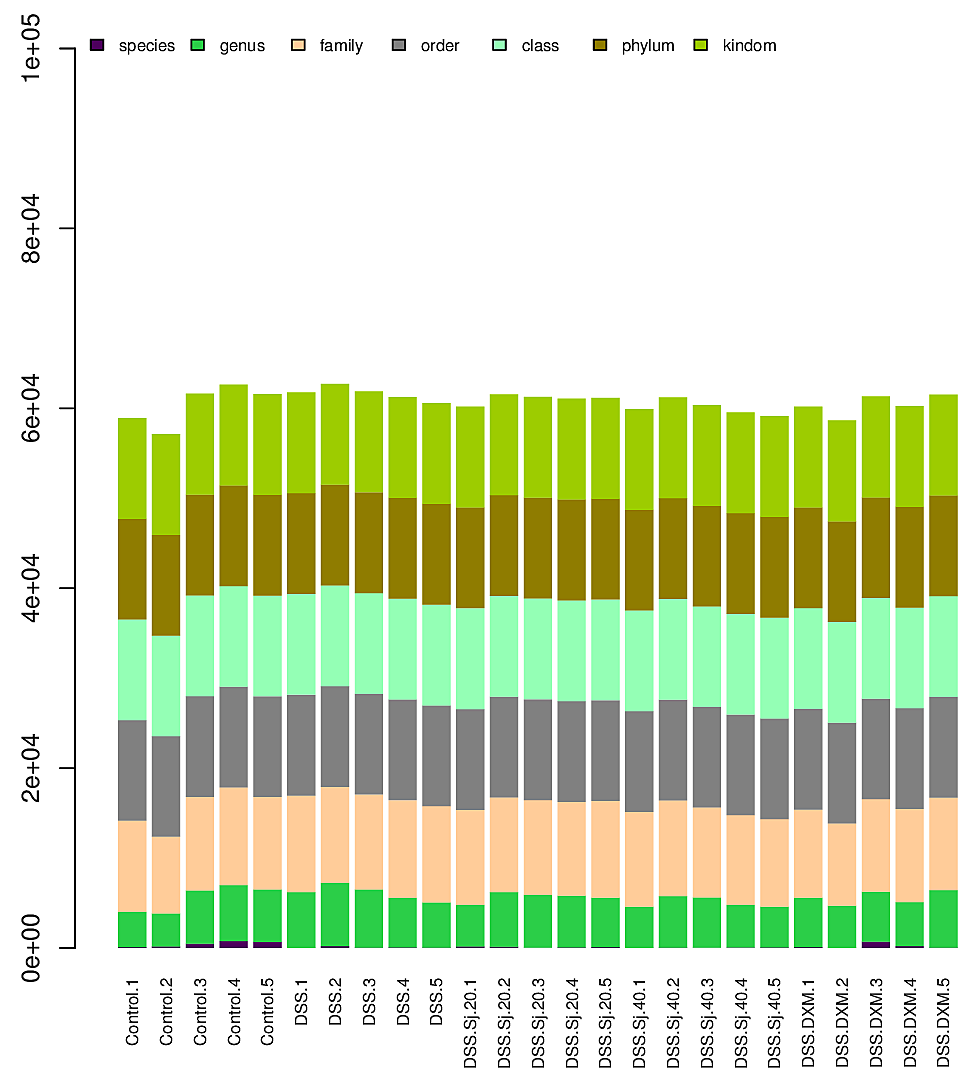

Supplement: Supplementary Figure 1 — The OTU level bar plot shows the summary of the total number of tags in the OTUs classified into each level (species, genus, family, order, class, phylum, and kingdom). [file Image_1.TIF]

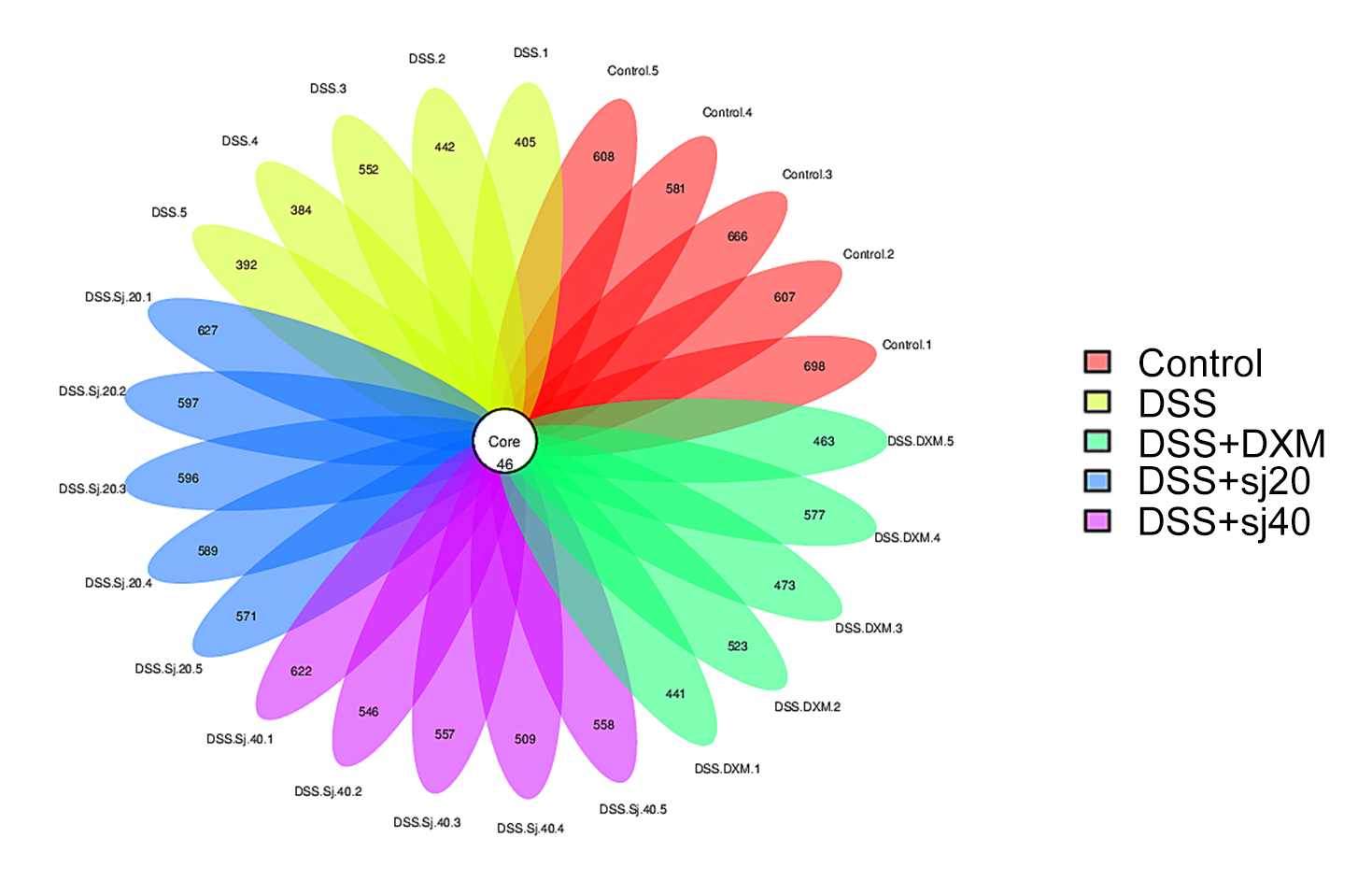

Supplement: Supplementary Figure 2 — The flower plot shows the unique and shared OTUs among all samples from the five groups. [file Image_2.TIF]

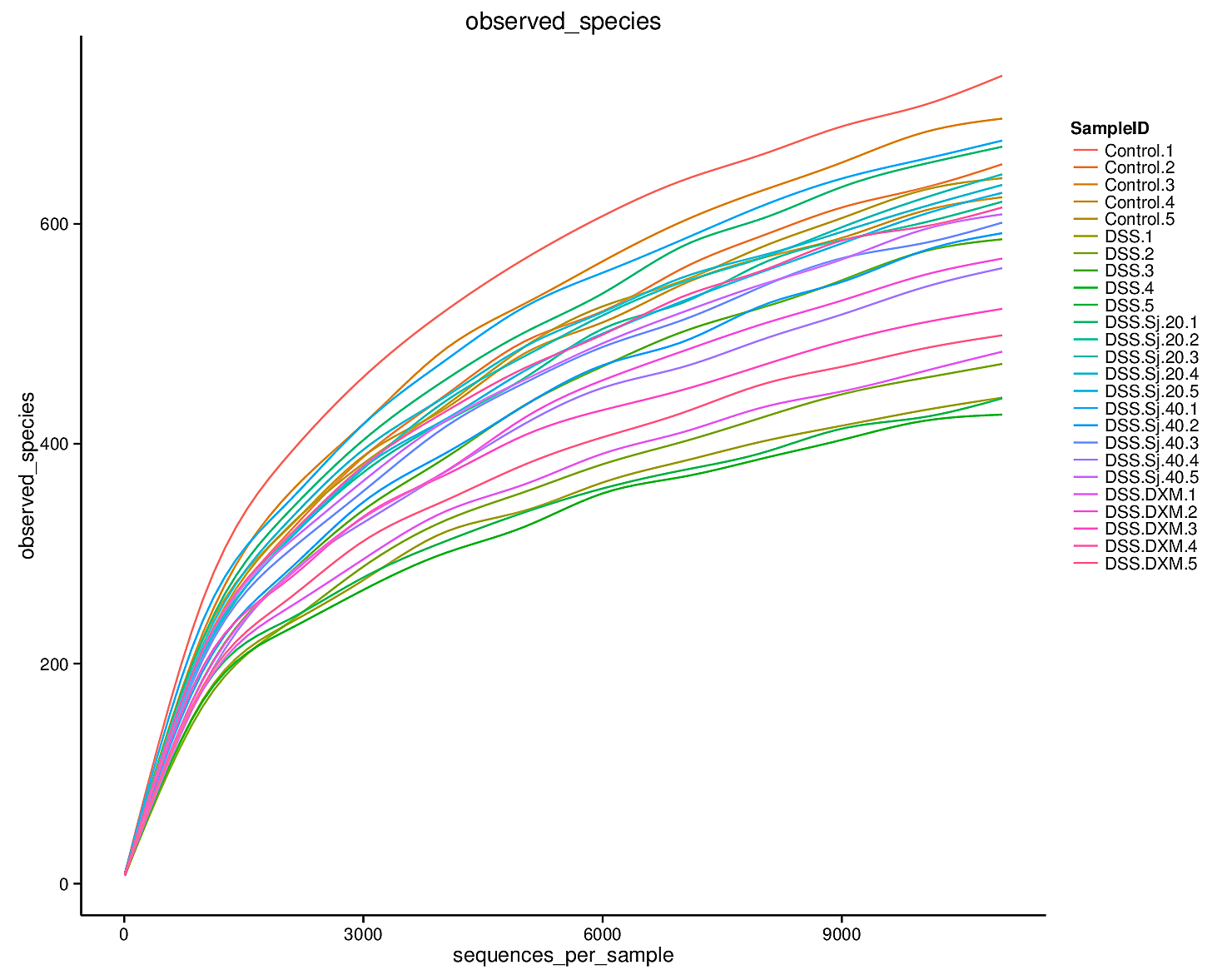

Supplement: Supplementary Figure 3 — Alpha diversity analyses of the observed_species reveal the richness of the gut microbiota. [file Image_3.TIF]

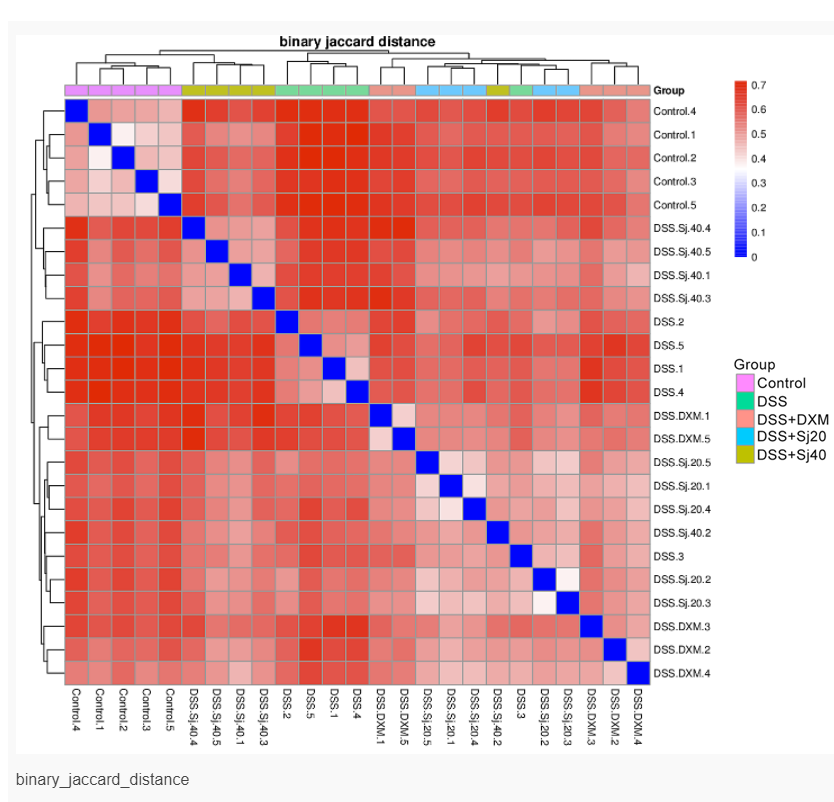

Supplement: Supplementary Figure 4 — Beta diversity analyses based on the hierarchical clustering method demonstrate the distance among the samples from the five groups. [file Image_4.TIF]

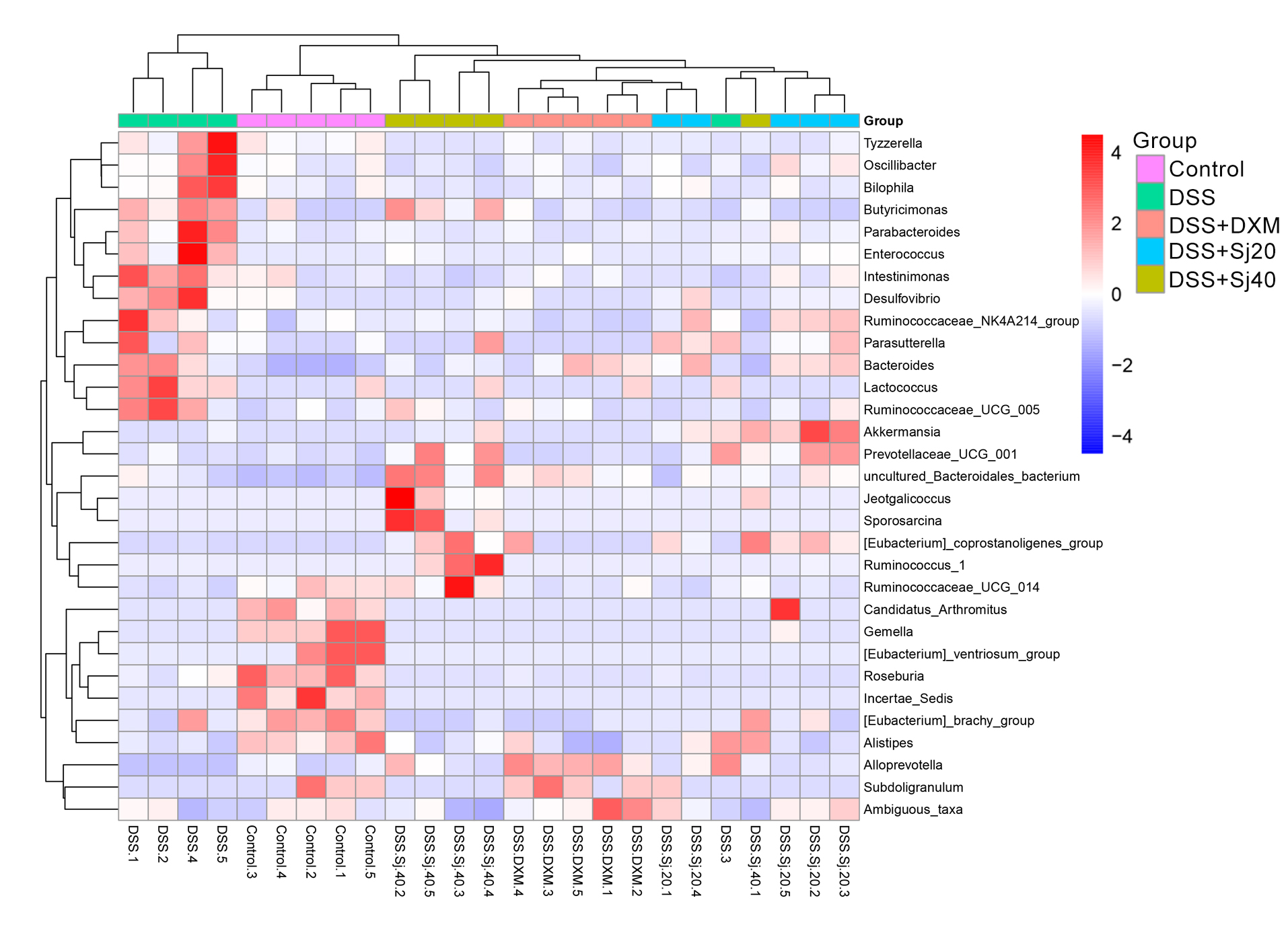

Supplement: Supplementary Figure 5 — The abundance of some bacterial genera regulated by the DSS + DXM and DSS + mSjci treatment were revealed by an ANOVA of genera. [file Image_5.TIF]

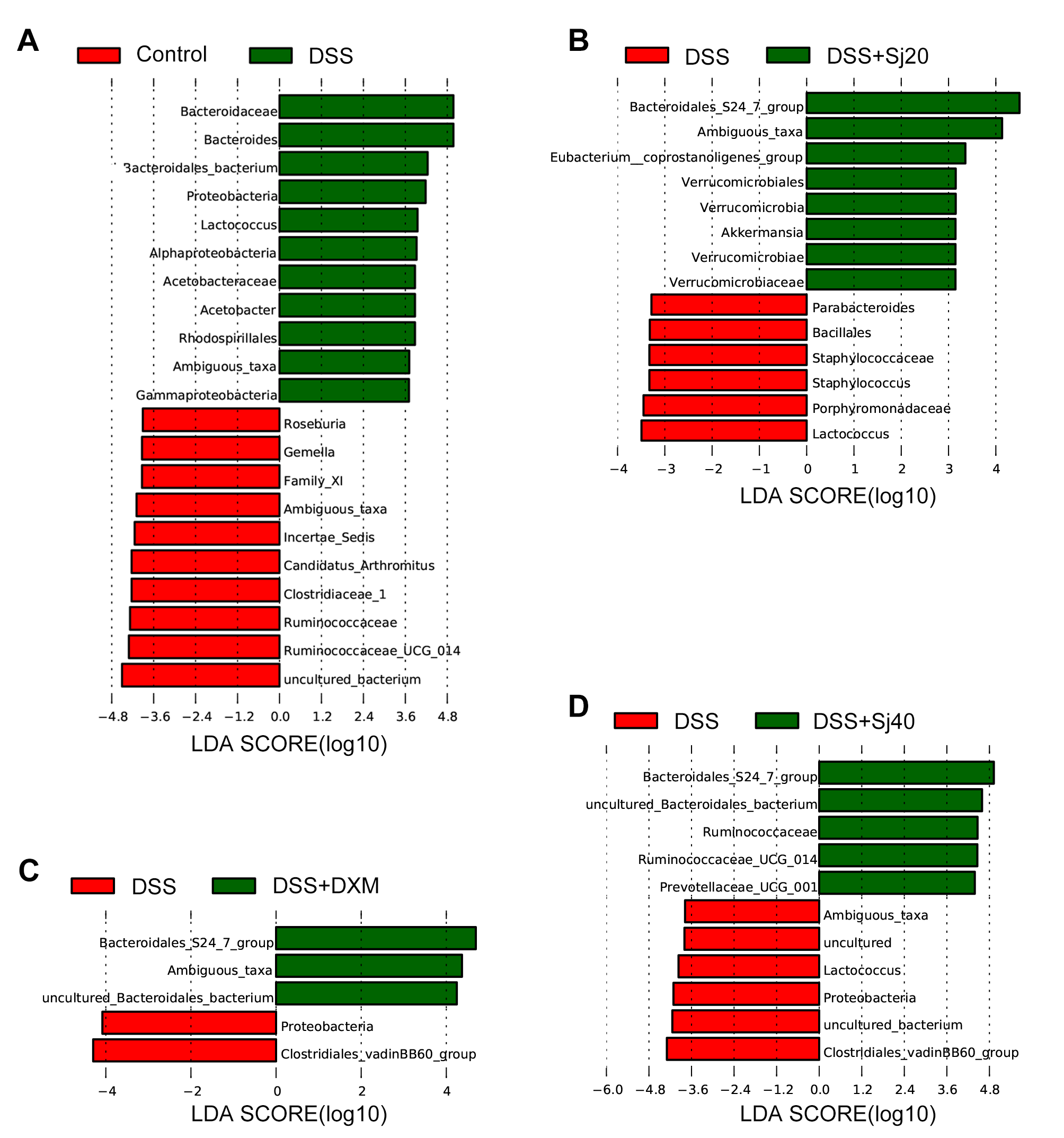

Supplement: Supplementary Figure 6 — The different genera between the four treatment groups and the DSS group show the significantly altered genera that are likely involved in the improvement of IBD. [file Image_6.TIF]
